# High Dose of Metformin Decreases Susceptibility to Occlusive Arterial Thrombosis in Diabetic Mice

**DOI:** 10.21203/rs.3.rs-3143156/v1

**Published:** 2023-07-11

**Authors:** Roberto I. Mota Alvidrez, Gowtham K. Annarapu, Amudan J. Srinivasan, Zeyu Liu, Hamza O. Yazdani, Richard L. Simmons, Sruti Shiva, Matthew D. Neal, Deidre Nolfi-Donegan

**Affiliations:** University of New Mexico; University of Pittsburgh; University of Pittsburgh Medical Center; University of Pittsburgh Medical Center; University of Pittsburgh Medical Center; University of Pittsburgh Medical Center; University of Pittsburgh; University of Pittsburgh Medical Center; University of Pittsburgh

**Keywords:** Metformin, arterial thrombosis, Diabetes, platelets, ADP

## Abstract

**Introduction:**

Metformin is the most prescribed medication in Diabetes Mellitus(DM). Metformin has shown to decrease mean platelet volume, with promising antiplatelet effects. High doses of Metformin have also been associated with hypercoagulation. We hypothesize that Metformin will protect DM mice from occlusive arterial thrombus formation by altering platelet activation and mitochondrial bioenergetics.

**Methods:**

DM was developed by low dose of Streptozotocin, healthy (non-DM) mice are controls. Either vehicle or Metformin was administered twice daily via oral gavage for 7-days. Ferric chloride (FeCl3) arterial thrombosis and tail bleeding time were performed. Whole blood aggregometry, platelet activation/adhesion and mitochondrial bioenergetics were evaluated.

**Results:**

Metformin decreased susceptibility of DM mice to arterial thrombosis. Platelet bioenergetics show DM mice have increased platelet mitochondrial respiration, but no differences were observed with Metformin treatment. In healthy mice, Metformin modulated ADP-dependent increase in platelet adhesion. In healthy mice, Metformin shortens bleeding time with faster thrombotic occlusion. Metformin also increased platelet mitochondrial maximal respiration and spare respiratory capacity uniquely in healthy mice.

**Conclusion:**

Metformin regulates platelet bioenergetics and ADP-mediated platelet function in DM mice which attenuates susceptibility to arterial thrombosis. Future studies will evaluate clinically relevant doses of Metformin that regulates thrombotic function in diabetic platelets.

## INTRODUCTION

Metformin is the most common first-line drug prescribed in DM patients(1). AMPKa is the main activated pathway targeted by antidiabetic therapeutics(2). AMPK is an established regulator of platelet function(3, 4). Higher doses of Metformin are prescribed to DM due to poor glucose control(5, 6). Same dose-dependent effect has been observed in murine models(7). Platelets from diabetic patients have pro-thrombotic susceptibility with distinct response to different platelet stimulants(8). High doses of Metformin (400mg/kg/day, this dose is routinely used in preclinical models) have shown to prevent arterial/venous thrombosis in diabetic rats by inhibiting extracellular mitochondrial DNA(9). Metformin has shown to affect platelet aggregation velocity and adhesion via ADP(10). Metformin has been uniquely linked with potential beneficial antiplatelet effects in gestational diabetes(11). Women with Gestational Diabetes received Metformin or placebo around 29 weeks of gestation. Soluble P-selectin was increased in the Metformin group with only a slight increase in soluble P-selectin in both placebo and Metformin from baseline(12).

The dose and frequency of Metformin that confers efficacy and safety to be used in multiple disease models is still to be determined(6). The use of standard doses of Metformin with combination therapies such as platelet inhibitors has not shown a synergistic benefit(13). Contrasting evidence has shown that similar high doses of Metformin are linked to treatment-related toxicity(14, 15). Contrasting findings are on high doses that alter coagulation cascade factors particularly by affecting liver function(16, 17).

Our study aims to test the effect of Metformin on the susceptibility to thrombosis in DM mice. The main hypothesis of our study is that high doses of Metformin will have a beneficial effect in decreasing susceptibility to occlusive thrombosis development in DM mice. Our findings illustrate the need for deeper studies of the beneficial antiplatelet dose- and time-dependent effects of Metformin in DM.

## MATERIALS AND METHODS

### Animal Studies

Metformin treatment was done for 7 days using oral gavage (317240-5GM, Millipore Sigma) twice daily at a dose of 200mg/kg per dose, autoclaved DI water was used as a treatment for controls (vehicle treated). Whole blood was collected after mice were sacrificed in collection tubes with 3.2% (0.109M) with sodium citrate in a 1:9 citrate:blood ratio (BD Biosciences).

### Low dose Streptozotocin (STZ) model of DM

C57/BL6 mice at 6 weeks of age were given 5-day low dose of STZ (572201-1GM, Millipore Sigma) IP 25mg/kg(18, 19). DM developed for 12 weeks. We performed body weight, 16hr fasting glycemia (75840-798, VWR) and insulin quantification via ELISA (ab277390, Abcam) in all mice.

### Ex vivo whole blood impedance aggregometry

Whole blood was diluted with saline (10:40 ratio) and treated with Metformin for 30 min at room temperature. Collagen at 1ug/mL of (Bio/Data^™^ 101562) or ADP (20uM) (Bio/Data^™^ 101312) were used as agonists. Data was collected using CHRONO-LOG^®^ Model 700 Aggregometer by measuring impedance and was analyzed using AGGRO/LINK^®^8 software.

### Platelet Bioenergetics

Platelet number was determined as described in Walkowiak et al.(20) Platelets (50 × 10^6^ per well) were loaded into an XF96 microplate with unbuffered Dulbecco’s Modified Eagle Medium (DMEM) and centrifuged at 1500g for 5 minutes. Platelets were treated with oligomycin A (2.5 μmol/L), carbonyl cyanide p-(trifluoro-methoxy) phenyl-hydrazone (FCCP; 0.7 μmol/L), and Rotenone (10 μmol/L). Oxygen consumption rate (OCR) was measured by XF analysis (21)(XF96, Seahorse Biosciences, Billerica, MA).

### Flow cytometry analysis of platelet activation

Platelet activation was measured in mouse whole blood (10:40 ratio) stained with anti-CD41a-FITC, anti-CD62P (P-selectin)-APC and JON/A antibody-PE (GPIIb/IIIa). Quantification was performed using BD LSR Fortessa^™^ flow cytometer with BD FACSDiva^™^ Software and analyzed with Flowjo 10.0. Positive anti-mouse CD41a-FITC was identified as platelet population. Activated platelets were quantified as positive gated on CD41 + population.

### FeCl3 injury and arterial thrombosis

Mice were anesthetized with 10mg/kg etomidate, 1.2g/kg urethane. After aseptic surgical area preparation, incision from the level of the manubrium to the mandible was made. Left carotid artery was isolated for placement of a doppler flow probe (Transonic Systems Inc., Ithaca, NY). A small plastic frit was placed under the carotid artery just proximal to the flow probe and a baseline flow reading was taken prior to placement of filter paper (Whatman) soaked with a fresh solution of 6% iron (III) chloride for 3 minutes. Surgical field was washed three times with PBS. Occlusive thrombosis was defined as cessation of flow without resumption over a 2-minute period in a 30-minute timepoint (5 seconds of zero-amplitude observed).

### Tail bleeding time

Anesthetized mice were placed in a supine position and 1cm of the tail tip was cut with a scalpel using a straight motion. Tail was submerged in a conical tube with 50mL of prewarmed (37°C) 0.9% Saline solution. Total bleeding time (sec) was recorded until no rebleed occurred within 3 min(22).

### Statistical Analysis

Results are presented as mean ± standard error. The number of biological samples and replicates are described in each assay. A sample size of 3–8 per experiment was used for each study, experiments were repeated 3 times. One-and two-way ANOVA was performed in GraphPad (Version 9.0.1). P-values < 0.05 were considered statistically significant.

## RESULTS

**Figure 1:**
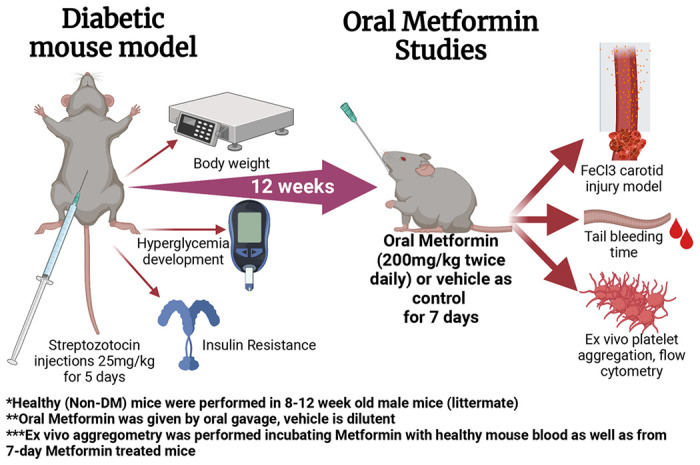
Diabetic mouse model development and oral Metformin studies methodologic scheme. Created with BioRender.

**Figure 2:**
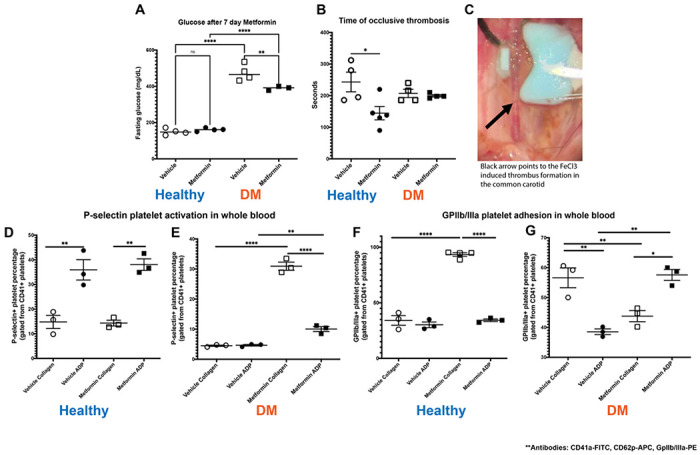
DM mice FeCl3 arterial thrombosis and flow cytometry analysis on 7-day oral Metformin (200mg/kg) treated mice. A) Effect of Metformin in vivo, decreased hyperglycemia in DM mice after 7 days with no effect in healthy mice(these mice did not exhibit hyperglycemia); student T-test B) Metformin shortens time to thrombus formation in normal mice while there is an apparent protective effect in DM mice; One way ANOVA: p 0.0258 with multiple comparisons. C) Representative image of FeCl3 surgical procedure with black arrow pointing to area of thrombus formation (white). D-E) Metformin increased platelet activation via P-selectin expression mostly with Collagen stimulation in DM mice after Metformin treatment; One-way ANOVA: p <0.0001 with multiple comparisons. F) In healthy mice Metformin increases platelet adhesion (GpIIb/IIIa expression) under Collagen stimulation with a minimal effect under ADP simulation; One-way ANOVA: p 0.0005 with multiple comparisons. G) In DM mice, Metformin decreased GpIIb/IIIa expression under Collagen stimulation with an inverse effect with ADP showing increase in GpIIb/IIIa expression; One-way ANOVA: p 0.0003 (G), p <0.0001 with multiple comparisons (H).

For our treatment groups, we either treated mice with Metformin or vehicle (control) for 7 days (Fig. 1). High dose of Metformin decreased hyperglycemia only in DM mice despite the short treatment duration (Fig. 2A). After 7-day treatment with Metformin, it was observed that DM mice did not have a difference in the time to FeCl3-induced occlusive thrombosis compared to vehicle treated mice (Fig. 2B–2C). However, in healthy mice treated with Metformin, thrombosis occurred more rapidly as demonstrated by a shorter time of occlusion (Fig. 2C) compared to vehicle treated healthy mice (Fig. 2B). In parallel, we performed flow cytometric analysis of markers of platelet activation and adhesion in both groups. We observed minimum effect in platelet activation in healthy mice (Fig. 2D) using flow cytometry analysis. DM mice showed increase in platelet activation, more importantly to Collagen when treated with Metformin (Fig. 2E). Baseline ADP platelet activation was lower in DM compared to healthy mice. Regarding platelet adhesion (GPIIb/IIIa expression), Metformin treatment in healthy mice increased GPIIb/IIIa with Collagen stimulation (Fig. 2F). DM mice show that Metformin decreased GPIIb/IIIa under collagen stimulation but increased under ADP stimulation (Fig. 2G).

**Figure 3:**
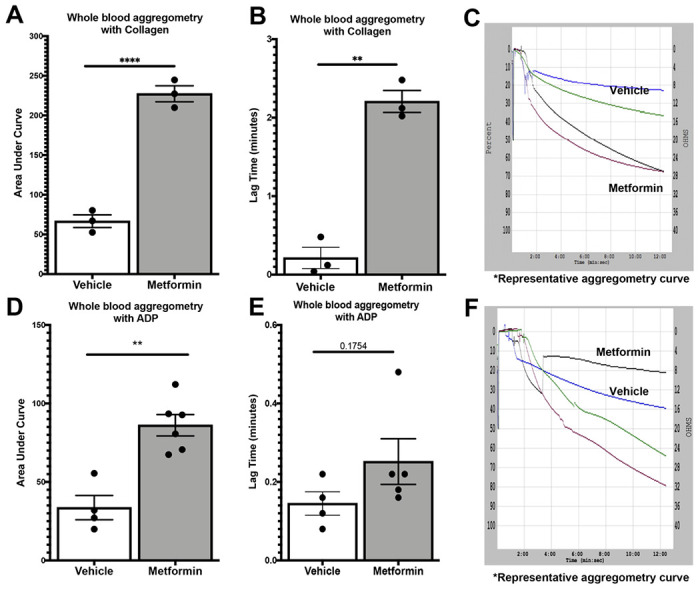
Whole blood 7-day oral Metformin (200mg/kg) aggregometry and flow cytometry with ADP/Ca+ and Collagen/Ca+ in healthy mice. A-C) Whole blood aggregometry with Collagen (1ug/mL). Data showed increased aggregation by AUC with Metformin treatment orally 200mg/kg twice daily compared to mice given vehicle for 7 days twice a day. Lag time is increased in Metformin treated mice; One-way ANOVA: p <0.0001 (A), p 0.0057 (B) with multiple comparisons. D-F) Whole blood aggregometry with ADP (20uM) showed increased aggregation by AUC in mice given Metformin orally 200mg/kg twice daily compared to mice given vehicle for 7 days twice a day. Paradoxically to our earlier findings in increased lag time in Metformin treated mice stimulated with Collagen, when we used ADP as an agonist for aggregometry lag time increase effect was lost; One-way ANOVA: p <0.0001 (D), p 0.1754 with multiple comparisons (E).

Because of all our previously described findings in healthy mice, we decided to further study the changes Metformin showed ex vivo by evaluating their platelet function. We assessed platelet aggregometry in healthy mice after 7-day treatment with Metformin compared to controls under both Collagen and ADP stimulation. There was a consistent increase in platelet aggregation (Fig. 3A) and lag time (Fig. 3B) in Metformin treated healthy mice regardless of length of stimulation (Fig. 3C). However, when we tested platelet aggregation using ADP, we found that Metformin also increased aggregation like Collagen (Fig. 3D) but there was no difference in lag time (Fig. 3E, 3F).

**Figure 4:**
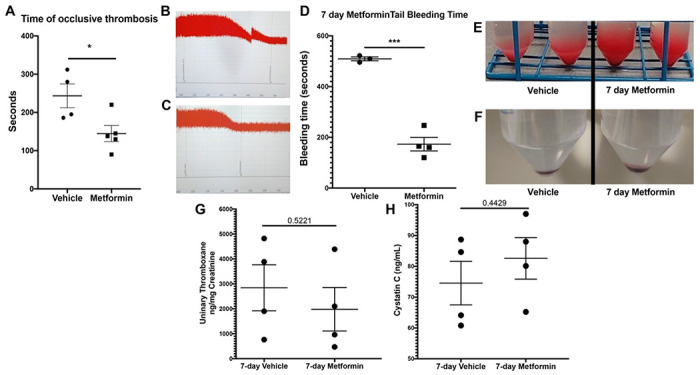
FeCl3 arterial thrombosis, bleeding time on 7-day Metformin treated mice. A) Metformin treatment results in faster time of occlusive thrombosis compared to vehicle treated mice*. B-C) Representative images of the readout of the time of occlusive thrombosis in the FeCl3 model in 7-day vehicle and Metformin treated mice. D) Oral Metformin treatment results in shorter tail bleeding time compared to vehicle treated mice. E-F) Representative images show amount of blood collected during tail bleeding assays and sediment of cells after centrifugation. G L-M) Platelet activation marker analysis with urinary Thromboxane and correlation with plasma Cystatin C levels for acute kidney injury development analysis showed no differences between vehicle and Metformin treated mice for 7 days. *Data was represented again in figure 2 to compare DM mice to healthy mice and is shown in this panel to show the effect of Metformin in normal mice.

We then move further in our analysis of healthy mice. As shown together with DM mice in Fig. 2, healthy mice exhibited shorter time of occlusive thrombosis when treated with Metformin (Fig. 4A–4C). In Fig. 4A we show the individual exposure plus representative traces of Metformin vs vehicle treated healthy mice. We also observed healthy mice have a shorter bleeding time when treated with Metformin compared to controls (Fig. 4D). However, healthy mice showed higher total blood/cells loss during that short time (Fig. 4E, 4F). Healthy mice had no development of kidney damage (Cystatin C) or associated changes in platelet activation evidenced by urinary thromboxane analysis (Fig. 4G, 4H).

**Figure 5:**
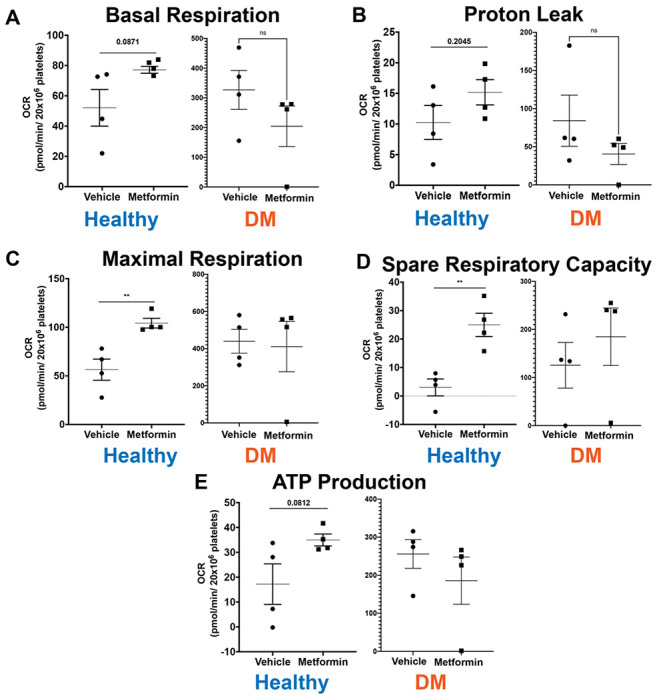
Platelet mitochondrial respiration after 7-Day Metformin Treatment. Due to chronic disease state due to diabetes, DM mice have higher threshold of platelet mitochondrial bioenergetics compared to healthy mice. Results show that DM mice do not evidence any differences in platelet bioenergetic parameters. Contrarily, healthy mice treated with Metformin show increase in maximal respiration and spare respiratory capacity. There also appeared to be an increase in basal respiration, proton leak and ATP production in Metformin treated healthy mice but there were no significant statistical differences found. Two-side t-test was performed: p<0.01 (C-D).

As a final evaluation of Metformin effect in platelet function, we evaluated platelet bioenergetics in both healthy and DM mice (Fig. 5). DM mice show higher baseline platelet mitochondrial respiration(23), consistent with hyperglycemia-induced increase in glucose metabolism(24). There were no differences with Metformin treatment in any of the parameters we interrogated in DM treated mice compared to controls (Fig. 5). These results are consistent with our protective effect of Metformin in DM of occlusive arterial thrombosis development. In contrast, high dose of Metformin increased maximal respiration and spare respiratory capacity in platelets from healthy mice compared to controls. All these findings correlate to the effect of Metformin in healthy mice showing shorter time of occlusive thrombosis and shorter bleeding time that is not observed in DM mice. We believe further studies of dose responses to Metformin are needed to show potential beneficial effects in disease(25,26)

## DISCUSSION

Our studies aim to better understand the effect of the commonly used high dose of Metformin treatment in diabetic arterial thrombosis. The novelty of our studies shows promising antiplatelet effects of commonly used preclinical dose of Metformin in arterial thrombosis. We have made striking observations on the distinct effects of Metformin in DM platelets, which remains an unexplored area. We used a published and proven model of DM(27, 28). We allow DM to develop for full 12 weeks before experimental studies. We started injections at 6 weeks of age and allowed an acclimation period after injections since the model is time- and STZ dose-dependent(18). We compared STZ injected mice (DM) to non-STZ injected (healthy) mice. There have been other studies done previously in diabetic platelets and arterial thrombosis(9, 29). However, we did find some differences in results compared to our studies. For example, Stolla et al in 2013 show results showing an effect in diabetic platelets in a model of FeCl3 arterial thrombosis. However, their results in the FeCl3 thrombosis model only present Mean Fluorescence Intensity quantification of Fibrinogen but not time of occlusion. Their mouse model was more indicative of a strong Type-1 Diabetes with less Insulin Resistance due to time (3 weeks vs 12 weeks) and the different dose of STZ used (50mg/kg vs 25mg/kg).

As for other limitations in our studies, we decided to test this dose of Metformin since promising effects had been observed in other small animal models of arterial thrombosis(9). Components of the coagulation cascade and thrombin activity could be affected by such high dose of Metformin that counterbalance potential beneficial effects in healthy mice(16, 30). However, those potential studies are beyond the scope of these studies but need to be further explored(31–33). We administered Metformin via oral gavage vs in drinking water which also increases the bioavailability and effect of Metformin(34). These factors could potentially explain why we observe such differences in healthy mice(35). We evidence almost no difference in time of occlusive thrombosis between healthy and DM mice. We believe that this is due to changes in lag time due to Metformin particularly in DM mice that are not evident in our 2-minute assay. Studies in other murine models of DM show that arterial thrombosis effects can be observed way beyond such cutoff(36, 37).

We believe that platelet activation results in Fig. 2 correlate AMPKa dependence in DM. Lack of significant AMPKa pathway activation in healthy as occurs in diabetes might explain changes in aggregation. There is evidence of platelet activation by increase in P-selectin in almost all our groups. However, the important evidence to highlight is that Metformin increased platelet adhesion via GPIIb/IIIa activation under Collagen stimulation in healthy mice. Contrarily, in DM mice, under Collagen stimulation, platelet adhesion is decreased. This would explain why DM mice treated with Metformin have less arterial thrombus formation as it mimics a physiologic condition in vivo even though there is platelet activation with Collagen. Since Metformin is an AMPKa activator, ADP effect could overshadow results since it is fundamental for the therapeutic effect of Metformin in diabetes(10, 38). Moreover, Metformin can replenish ATP levels by increasing ATP/ADP ratio particularly under hyperglycemic conditions(39). This concludes that the effect of Metformin might be difficult to mimic of the altered platelet responses in vivo that we are not being able to replicate exactly ex vivo(40). However, there is benefit in reducing platelet specific effects in a dose-dependent manner of Metformin but apparently is unique to DM patients(7).

As summary, Metformin decreases susceptibility to develop occlusive arterial thrombosis only in DM mice. We demonstrate an unexpected Metformin-induced increased propensity for thrombosis in healthy mice. Further studies will be needed to underpin the mechanisms by which dose-dependent effects of Metformin mediate ADP-regulated platelet function in DM.

## Data Availability

The data that support the findings of this study are available in the MENDELEY DATA repository, XXX
